# Chemotherapy induces the cancer-associated fibroblast phenotype, activating paracrine Hedgehog-GLI signalling in breast cancer cells

**DOI:** 10.18632/oncotarget.3828

**Published:** 2015-04-14

**Authors:** Maria Peiris-Pagès, Federica Sotgia, Michael P. Lisanti

**Affiliations:** ^1^ The Breakthrough Breast Cancer Research Unit, Institute of Cancer Sciences, University of Manchester, UK; ^2^ The Manchester Centre for Cellular Metabolism (MCCM), Institute of Cancer Sciences, University of Manchester, UK

**Keywords:** chemotherapy, metabolism, second primary tumours, tumour microenvironment

## Abstract

Cancer cells recruit normal cells such as fibroblasts to establish reactive microenvironments. Via metabolic stress, catabolism and inflammation, these cancer-associated fibroblasts set up a synergistic relationship with tumour cells, that contributes to their malignancy and resistance to therapy. Given that chemotherapy is a systemic treatment, the possibility that healthy cell damage affects the metastatic risk or the prospect of developing a second malignancy becomes relevant. Here, we demonstrate that standard chemotherapies phenotypically and metabolically transform stromal fibroblasts into cancer-associated fibroblasts, leading to the emergence of a highly glycolytic, autophagic and pro-inflammatory microenvironment. This catabolic microenvironment, in turn, activates stemness (Sonic hedgehog/GLI signalling), antioxidant response and interferon-mediated signalling, in adjacent breast cancer cells. Thus, we propose a model by which chemotherapy-induced catabolism in healthy fibroblasts constitutes a source of energy-rich nutrients and inflammatory cytokines that would activate stemness in adjacent epithelial cells, possibly triggering new tumorigenic processes. In this context, immune cell recruitment would be also stimulated to further support malignancy.

## INTRODUCTION

**Figure 1:**
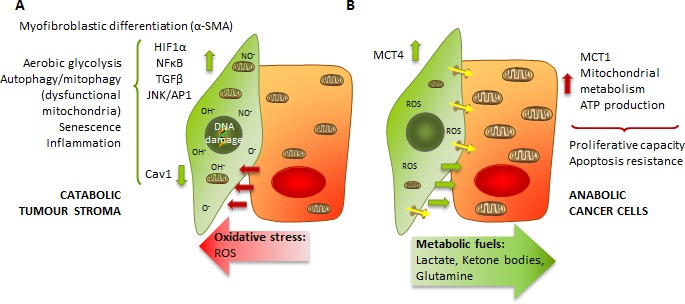
The catabolic tumour stroma phenotype **A.** ROS production by rapidly proliferating cancer cells generates oxidative stress to surrounding stromal cells, which induces changes in them such as CAF transformation, activation of HIF1, NFkB, TGFβ or JNK/AP1 signalling pathways, a switch to aerobic glycolysis and mitochondrial dysfunction, autophagy and senescence and release of inflammatory cytokines: the catabolic tumour stroma phenotype. **B.** Due to increased glycolysis and autophagy, there is a higher production of energy-rich metabolites such as lactate that are secreted by stromal cells and taken up by cancer cells, which use them to fuel their mitochondrial metabolism and ATP production.

Cancer cells are known to be the engine that drives tumour development, although it is now evident that they require help. Endothelial cells, immune cells, adipocytes or fibroblasts are recruited to establish tumorigenic microenvironments, which cooperate with cancer cells to enable and support most of their hallmarks [1]. Stromal fibroblastic cells infiltrate and are induced to differentiate into cancer associated fibroblasts (CAFs) [2], which contribute to the energetics of tumour cells by fuelling them with energy sources such as lactate, ketones, glutamine, fatty acids or cysteine, reinforcing not only their metabolic efficiency and growth but also their survival [3-6] (Figure 1A-1B). Likewise, through lactate-induced extracellular acidification and secretion of inflammatory cytokines and matrix-degrading enzymes, CAFs enable metastasis and confer resistance to anti-tumour drugs [3, 7, 8].

The aim of conventional therapy is to specifically kill the malignant cells within a tumour, although it can also damage normal tissues and the tumour microenvironment. Indeed, secretion of factors associated with inflammation and cancer progression has been detected in therapy-damaged senescent fibroblasts, suggesting that current cancer treatments may contribute to metastasis and *de novo* tumorigenesis [9]. One of the most severe side-effects of cancer treatment is actually the growth of a second primary tumour, an entirely new malignancy different from metastatic growth. Second primary cancers already account for one in six new cancer diagnoses in the USA and are a significant cause of mortality amongst patients who have been cured of cancer, being the leading cause of death in Hodgkin Lymphoma survivors [10]. Increased risk of developing a second malignancy has been reported after treatment with either radiotherapy or chemotherapy agents, including alkylating agents, topoisomerase inhibitors and anti-metabolites, and it is dose-dependent [10]. Nevertheless, high radiation doses concentrated on a limited field are less tumorigenic than lower doses exposed to a wider area [11]. Hence, considering the systemic nature of chemotherapeutic, the influence of therapy-damaged non-target cells on the risk of developing a subsequent malignancy becomes significant.

In this study, we test the hypothesis that chemotherapy promotes the same responses in human fibroblasts as their interaction with cancer cells, the so-called catabolic tumour stroma phenotype, which would constitute an ideal environment for a new neoplastic lesion to develop. We provide *in vitro* evidence of treatment-induced modifications in fibroblastic cells including the expression of αSMA, a glycolytic switch, an activation of the JNK/AP1, HIF1, TGFβ/SMAD, STAT3 and NFkB stress-induced pathways, senescence and autophagy, and a greater secretion of the inflammatory cytokine interleukin 6 (IL6). Finally we show the induction of several stemness, antioxidant and immunogenic signalling pathways in breast cancer cells when co-cultured with stromal fibroblasts in response to chemotherapy.

## RESULTS

To study the effects of chemotherapy on stromal cell behaviour, human fibroblasts were treated with different drugs and the induction of the catabolic tumour stroma phenotype was assessed.

We first hypothesised that chemotherapy would increase lactate and ketone production, as we previously showed their tumourigenic and metastatic effects *in vivo* [12]. As a first screening, L-lactate and β-hydroxybutyrate (β-HB) concentration was measured in hTERT-BJ1 fibroblasts after treatment with 12 different commonly used drugs (Table 1) including azathioprine, carboplatin, cisplatin, cyclophosphamide, doxorubicin, 5-fluorouracil, gemcitabine, methotrexate, 6-mercaptopurine, mitoxantrone, 6-thioguanine and taxol, detecting an increase in their production of L-lactate or β-HB compared to vehicle (Table S1-S2). Six agents were selected to proceed with the study according to their nature and their potential on increasing L-lactate and β-HB production at concentrations lower than 1 mM. Azathioprine (AZA), an anti-metabolite and carboplatin (CP) and cisplatin (CIS), both alkylating-like agents, were chosen and used for further studies at a concentration of 100 μM. Likewise, doxorubicin (DOX) and mitoxantrone (MTX), both topoisomerase inhibitors and taxol (TAX), a cytoskeleton drug, were selected at 100 nM. All chosen concentrations were sub-lethal and caused a decrease in cell viability lower than 50% after 72 h of treatment (Figure S1).

**Table 1:** Chemotherapeutic agents used in the current study

Chemotherapy dmg (Abbreviation)	Company (Reference)	Type
**Azathoiprine (AZA)**	**Sigma (A4638)**	**Anti-metabolite**
**Carboplatin (CP)**	**Sigma (C7_538)**	**Allcylating / alkylating agent**
**Cisplatin (CIS)**	**Sigma (C2210000)**	**Alkylating / alkylating agent**
Cyclophosphamide (CPA)	Sigma (C0768)	Alkylating/ a lkylat ing went
**Doxorubicin (DOX)**	**Sigma (D2975000)**	**Topoisomerase II inhibitor**
5-Fluorouracil(5-FU)	Sigma (66627)	Anti-metabolke
Gemcitabine (GC)	Sigma (G6423)	Anti-metabolke
Methotrexate (MET)	Sigma (M.29)	Anti-_metabolke_
6-Mercaptopurine(6-MP)	Sigma (852678)	Anti-metabolke
**Mitoxantrone (MIX)**	**Sigma (M6545)**	**Topoisomerase II inhibitor**
6-Th i °guanine (6-TG)	Sigma (A4660)	Anti-metabolke
**Taxol or paclitaxel (TAX)**	**Sigma (Y0000698)**	**Cytoskeleton drug**

### Chemotherapy transforms stromal fibroblasts in highly glycolytic, less metabolically active cells

**Figure 2:**
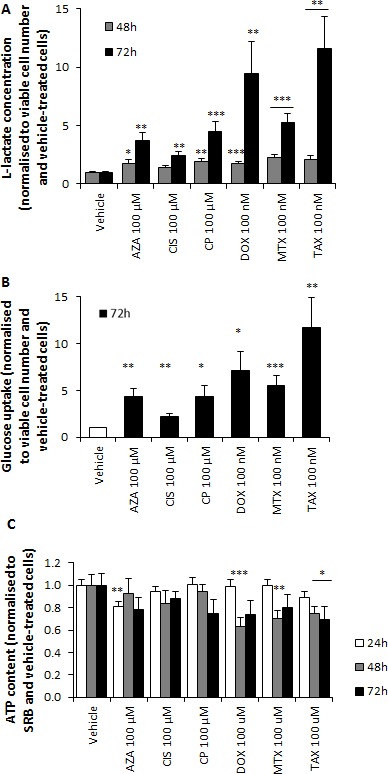
Chemotherapy increases L-lactate production and glucose consumption and reduces ATP content of hTERT-BJ1 fibroblasts **A.** Chemotherapy increases L-lactate production. L-lactate secretion by hTERT-BJ1 fibroblasts treated with chemotherapy for 48 and 72 h normalised to vehicle-treated cells. All drugs increased lactate production. Mean ± SEM. **B.** Chemotherapy increases glucose consumption. Glucose consumption by hTERT-BJ1 cells treated with chemotherapy for 72 h normalised to vehicle-treated cells. All drugs augmented glucose uptake. Mean ± SEM. **C.** Chemotherapy reduces ATP content. ATP content in hTERT-BJ1 cells treated with chemotherapy for 24, 48 and 72 h normalised to vehicle-treated cells. Azathioprine, doxorubicin, mitoxantrone and taxol decreased the ATP levels at some of the assessed time points. Mean ± SEM.

**Figure 3:**
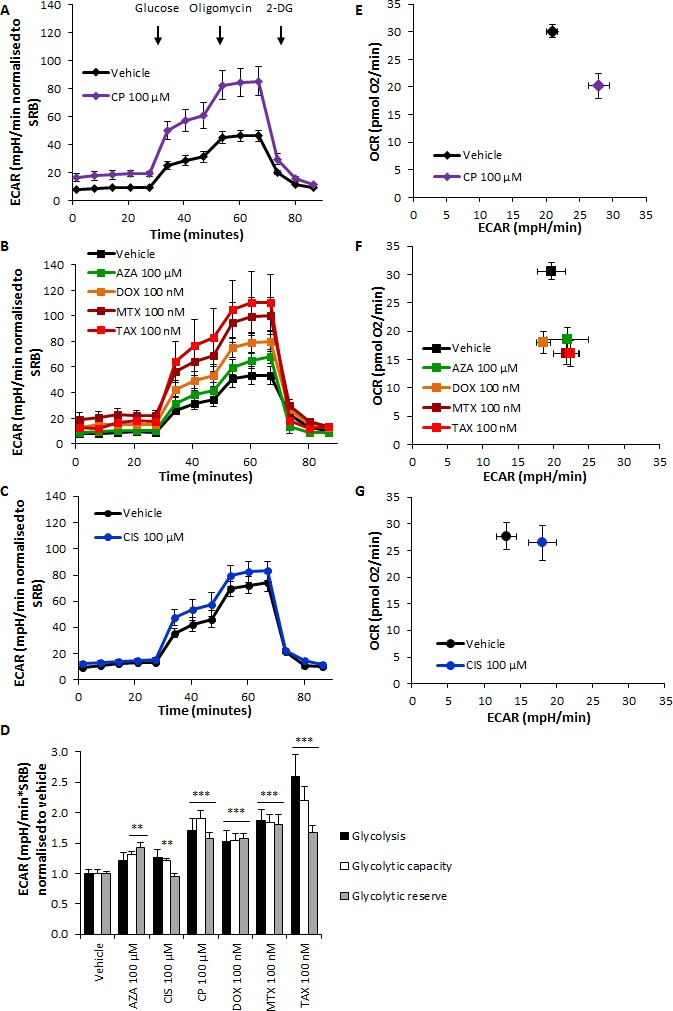
Chemotherapy increases glycolytic function of hTERT-BJ1 fibroblasts and transforms them into less metabolically efficient cells **A.** hTERT-BJ1 fibroblasts were treated for 72 h with carboplatin, **B.** azathioprine, doxorubicin, mitoxantrone, taxol and **C.** cisplatin before the extracellular efflux assay was performed. All drugs increased the ECAR of hTERT-BJ1 cells throughout the experiment compared to vehicle treatments. Mean ± SEM. **D.** Glycolysis, glycolytic capacity and glycolytic reserve measurements of chemotherapy-treated hTERT-BJ1 fibroblasts relative to vehicle-treated cells. All drugs significantly increased glycolytic capacity, and most of them also augmented the glycolytic reserve of hTERT-BJ1 fibroblasts (except cisplatin). Likewise, basal glycolytic rate was enhanced in carboplatin, doxorubicin, mitoxantrone and taxol treatments. Mean ± SEM. **E.** hTERT-BJ1 fibroblasts were treated for 72 h with carboplatin, **F**. azathioprine, doxorubicin, mitoxantrone, taxol and **G**. cisplatin before the extracellular efflux assay in the presence of glucose was performed, and the OCR versus ECAR graph was represented. All drugs induced a shift towards a less metabolically active phenotype in hTERT-BJ1 cells. Mean ± SEM.

High lactate levels suggested a chemotherapy-induced glycolytic switch in stromal fibroblasts. Indeed, L-lactate production and glucose consumption were significantly higher in all treatment conditions compared to vehicle (Figure 2A-2B), and ATP content was lower after azathioprine, doxorubicin, mitoxantrone and taxol treatments (Figure 2C). The extracellular acidification rate (ECAR) was subsequently measured using an XF96 Extracellular Flux Analyzer (Figures 3A-3C and S2A) and glycolysis, glycolytic capacity and glycolytic reserve were calculated after addition of glucose, oligomycin and 2-DG into the media. All drugs significantly increased glycolytic capacity and most of them augmented the basal glycolytic rate and glycolytic reserve (Figure 3D). Oxygen consumption rate (OCR) and ECAR were also measured in media containing glucose to plot the OCR versus ECAR graph, which indicates the metabolic state of the cell (Figure S2B). All agents notably induced a switch from a more aerobic and metabolically active phenotype towards a more glycolytic, less metabolically active phenotype (Figure 3E-3G).

**Figure 4:**
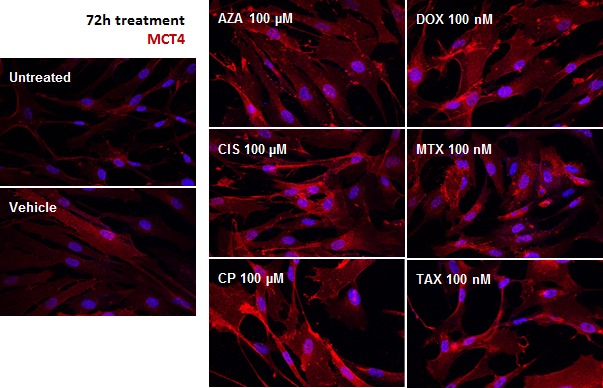
MCT4 expression is up-regulated in response to chemotherapy Representative pictures of hTERT-BJ1 fibroblasts treated for 72 h and immunostained for MCT4 (red). Nuclei were counterstained with DAPI (blue). Untreated and vehicle-treated hTERT-BJ1 images are also shown.

Increased ECAR indicated that the lactate produced by fibroblasts upon treatment was being secreted. Indeed, the expression of MCT4, the transporter that mediates monocarboxylate efflux, was perceptibly up-regulated in chemotherapy-treated hTERT-BJ1 compared to vehicle, as observed by immunofluorescence (Figure 4). Thus, chemotherapy stimulates fibroblasts to consume more glucose and produce more lactate, which is released via enhanced MCT4 expression, hence increasing extracellular acidification. According to the less efficient ATP production during glycolysis than in complete oxidative phosphorylation, the ATP content is minor.

### Chemotherapy promotes oxidative stress and stress-induced signalling pathways in stromal fibroblasts

**Figure 5:**
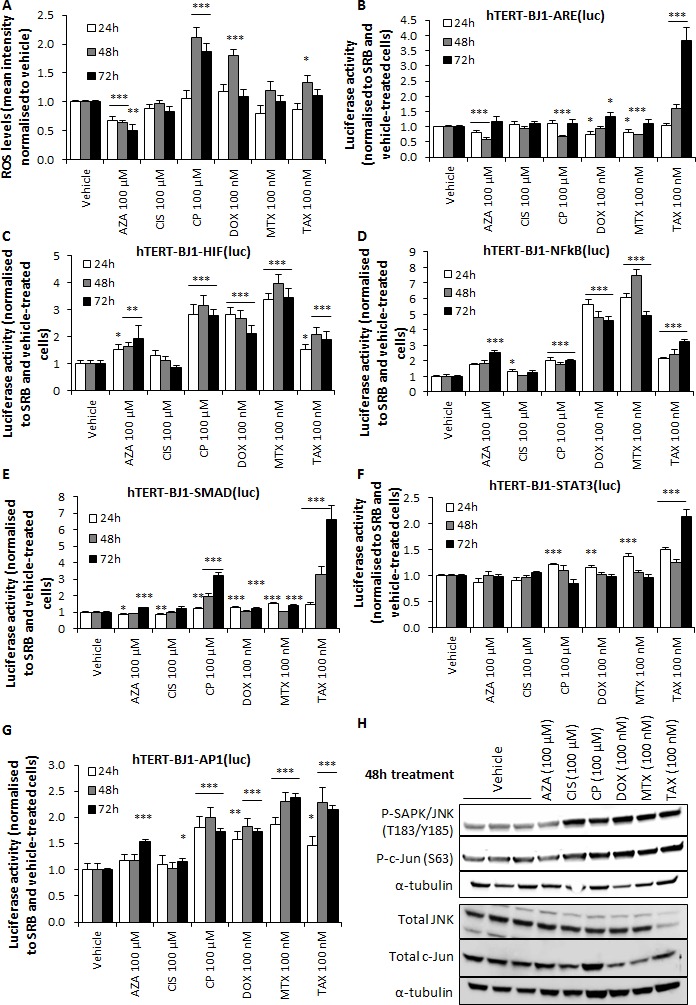
Chemotherapy induces oxidative stress and activates stress signalling pathways in hTERT-BJ1 fibroblasts **A.** Intracellular ROS levels of hTERT-BJ1 fibroblasts treated with chemotherapy for 24, 48 and 72 h normalized to vehicle-treated cells. At 48 h of chemotherapeutic treatment, carboplatin, doxorubicin and taxol significantly induced the production of ROS. Mean ± SEM. **B.** ARE-dependent signalling pathway activation after treatment with chemotherapy for 24, 48 and 72 h normalised to SRB and vehicle-treated cells. Only doxorubicin and taxol were able to significantly activate Nrf1 and Nrf2-mediated antioxidant response at late time points. Mean ± SEM. **C.** Significant activation of HIF **D.** NFkB, **E.** SMAD, **F.** STAT3 and **G.** AP1 signalling pathway in response to chemotherapy after 24, 48 and 72 h normalised to SRB and vehicle-treated cells. All drugs were able to activate NFkB and AP1. All drugs except cisplatin could also increase HIF and SMAD activation. Likewise, all drugs except azathioprine and cisplatin significantly stimulated STAT3 24 h after treatment. Mean ± SEM. **H.** Chemotherapy-induced increase in the phosphorylation levels of SAPK/JNK and c-Jun in hTERT-BJ1 fibroblasts as measured by Western blotting after 48 h of treatment with all drugs except azathioprine. Total JNK and c-Jun protein levels did not show substantial differences. Only carboplatin intensified total c-Jun protein expression levels.

We next sought to determine the intracellular ROS levels of chemotherapy-treated hTERT-BJ1 fibroblasts, as chemotherapy has been shown to cause oxidative stress in cancer cells and healthy tissues [13, 14]. Carboplatin, doxorubicin and taxol treatments enhanced ROS production, whereas cisplatin and mitoxantrone did not modify ROS levels and azathioprine reduced them (Figure 5A). To evaluate whether high oxidative stress was due to an antioxidant response deficiency, we generated hTERT-BJ1-ARE(luc) reporter cells, which were exposed to chemotherapy and assessed for luciferase activity. Taxol and doxorubicin induced a significant activation of luciferase activity at late time points. Other drugs did not increase or even significantly reduced ARE-dependent response, including carboplatin, which produced the highest ROS levels (Figure 5B). Thus, some drugs were able to induce oxidative stress in stromal fibroblasts at the chosen concentrations, but either failed to elicit antioxidant response or that response was slow, implying a lack of a strong antioxidant response as one of the reasons behind the increase in ROS production.

Oxidative stress in the stroma, which is generally caused by cancer cells at the onset of their synergy, promotes CAF transformation and HIF1, NFkB, JNK, STAT3 and TGFβ activation in the stromal compartment [6, 15, 16]. Thus, we next assessed the activation of these signalling pathways. Five reporter hTERT-BJ1 cell lines were generated (Table 2), exposed to chemotherapy, and luciferase activity was determined. All drugs significantly amplified NFkB and AP1 signalling (Figure 5D, 5G). Likewise, HIF, SMAD and STAT3 signalling was increased by most agents (Figure 5C, 5E-5F). Finally, upstream of AP1, the phosphorylation levels of SAPK/JNK and c-Jun were also up-regulated after all drug treatments except azathioprine relative to vehicle-treated fibroblasts, as measured by immunoblotting, whereas total JNK and c-Jun protein levels did not show substantial differences, indicating an activation of the whole JNK/AP1 pathway in chemotherapy-treated hTERT-BJ1 fibroblasts. Carboplatin also intensified total c-Jun protein expression levels (Figure 5H).

**Table 2:** Cignal Lenti reporter assay and GFP vectors used in this study and cell line utilised for transduction for each of them

Vector	Genecopoeia (reference)		Cell line	Selection
GFP	EX-ECFP-Lv1S1		MCF7	G418 1 mg/ml
**Cignal Lenti reporter assay (luc)**	**Qiagen (reference)**	**Pathway**	**Cell line**	**Selection**
AP1(luc)	CLS-011L	JNK/AP1-regulated signaltransduction	hTERT-S.11	Puromycin2 kg/m1
APE(luc)	CLS-20201	Nrf2- and Nrfl-mediated antioAdant response	hTERT-BJ1 and MCF7-GFP	Puromycin2 *gglml*
GAS(luc)	CLS-009L	Interferongamma-induced signal transducdon	hTERT-9.11 and MCF7-GFP	Pu romyc in 2 ug/m1
GLI(luc)	CLS-3030L	Hedgehog signaling pathway	MCF7-GFP	Puromycin 2 pg/m1
HIF(luc)	CLS-007L	HIF-mediated hypoxia signaling	hTERT-9.11	Puromycin 2 pg/ml
ISRE(luc)	CLS-008	Type I interferon-induced signal transduction	hTERT-911 and MCF7-GFP	Puromycin2 Wm!
NFRB(Iuc)	CLS-0131	NFKB-regulated signal transduction	hTERT-B.11	Pu romyc in 2 p,g/m1
SMAD(luc)	CLS-0171	TGF9-induced signal transduction	hTERT-BJ1 and MCF7-GFP	Puromycin 2 pg/m1
STAT3(luc)	CLS-60281	Transcriptional activity of STAT3	hTERT-13.11 and MCF7-GFP	Pu romycin 2 og/m1
TCF/LEF(luc)	CLS-018L	Wnt signal transduction	MCF7-GFP	Puromycin2 isgtml

### Chemotherapy triggers autophagy, senescence, and IL6 secretion in stromal fibroblasts

**Figure 6:**
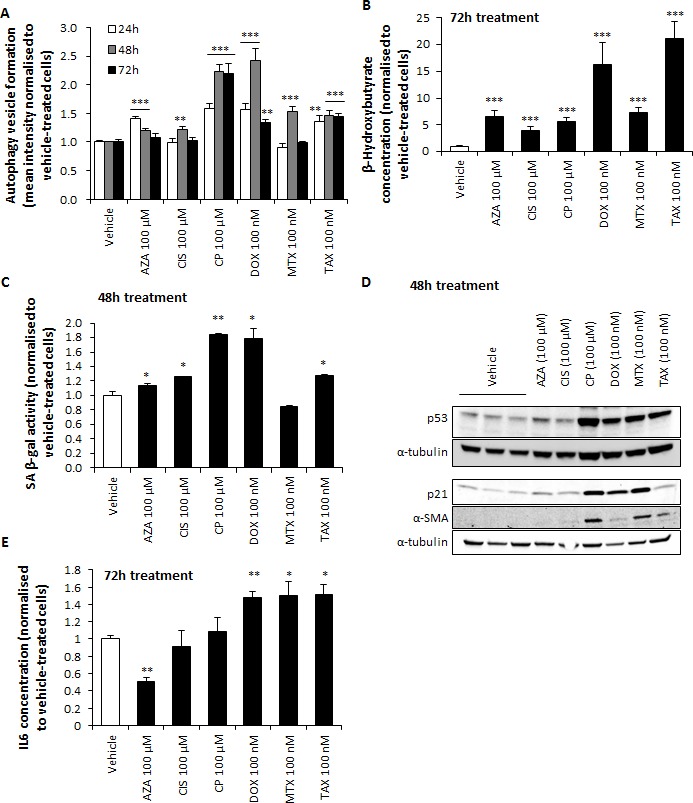
Chemotherapy induces autophagy, senescence, CAF transformation and IL6 secretion in hTERT-BJ1 fibroblasts **A.** Autophagy vesicle staining in hTERT-BJ1 fibroblasts after 24, 48 and 72 h of treatment with chemotherapy normalised to vehicle-treated cells. All drugs significantly increased autophagy at all or some of the assessed time points. Mean ± SEM. **B.** β-HB secretion by hTERT-BJ1 fibroblasts after 72 h of chemotherapy treatment normalised to vehicle. All drugs significantly increased β-HB concentration. Mean ± SEM. **C.** Quantification of SA-β-gal activity as measured by FDG-fluorescein staining in hTERT-BJ1 fibroblasts after 48 h of chemotherapy treatment normalised to vehicle-treated cells. All drugs except mitoxantrone significantly increased the levels of SA-β-gal. Mean ± SEM. **D.** Chemotherapy-induced up-regulation of p53, p21 and α-SMA in hTERT-BJ1 fibroblasts as measured by Western blotting. All drugs except azathioprine and cisplatin clearly increased p53 and α-SMA expression. Expression of p21 was also found up-regulated in all drugs except cisplatin and taxol. **E.** Interleukin 6 levels present in media of hTERT-BJ1 fibroblasts treated for 72 h with chemotherapy as measured by ELISA. Doxorubicin, mitoxantrone and taxol significantly increased IL6 secretion. Mean ± SEM.

Autophagy and senescence are known to be a common response to DNA-damaging agents such as chemotherapy [17, 18]. Autophagy is a process by which lipid degradation is induced and therefore ketone bodies are generated. Thus, we decided to assess whether chemotherapy can also induce autophagy and ketogenesis in these cells. Stromal fibroblasts were exposed to chemotherapy and subsequently stained for autophagic vacuole production, which was significantly enhanced by all drugs (Figure 6A). We next measured the levels of ketones in the media of chemotherapy-treated fibroblasts, and in line with our first screening we found that all drugs induced β-HB secretion (Figure 6B). To determine senescence levels after chemotherapy treatment, hTERT-BJ1 fibroblasts were assessed for senescence-associated β-galactosidase (SA-β-gal) activity, which appeared to be significantly greater in all drug treatments except mitoxantrone (Figure 6C). Alternatively, expression of p53 and p21, known regulators of cellular senescence, was also assessed by immunoblotting. Most drugs showed p53 and p21 over-expression (Figure 6D). Collectively, these results indicate higher autophagy and senescence in hTERT-BJ1 fibroblasts upon treatment.

One of the features of senescence is the tumour-promoting senescence-associated secretory phenotype (SASP), which occurs in response to treatment-induced DNA damage in normal and tumour cells [19, 20] and contains inflammatory cytokines, which can further aggravate chemotherapy-related inflammation. We therefore decided to determine whether chemotherapy would induce secretion of inflammatory cytokines by screening the media of hTERT-BJ1 cells treated with either doxorubicin or vehicle using a Cytokine Antibody Array. IL6 was the most up-regulated cytokine in doxorubicin-treated fibroblasts relative to vehicle (Figure S3A). Indeed, amongst SASP-secreted cytokines there is IL6, which is also present in the media of fibroblasts and cancer cells in co-culture [21]. Doxorubicin, mitoxantrone and taxol significantly increased the secretion of IL6 in hTERT-BJ1 fibroblasts, as confirmed by ELISA, whereas azathioprine decreased it (Figure 6E).

### Chemotherapy induces myofibroblastic transformation

Myofibroblasts or CAFs are abundant components of the tumour microenvironment and mostly accountable for the development of fibrosis, a common side effect of cancer therapy. CAFs are characterised by the expression of α-smooth muscle actin (αSMA) [22]. Therefore, we determined αSMA expression on lysates of chemotherapy-treated hTERT-BJ1 fibroblasts, observing an up-regulation of αSMA by most drugs (Figure 6D). Thus, chemotherapy can trigger transformation of fibroblasts into myofibroblasts.

### Upon treatment, stromal fibroblasts stimulate stemness, antioxidant and immune response in breast cancer cells

**Figure 7:**
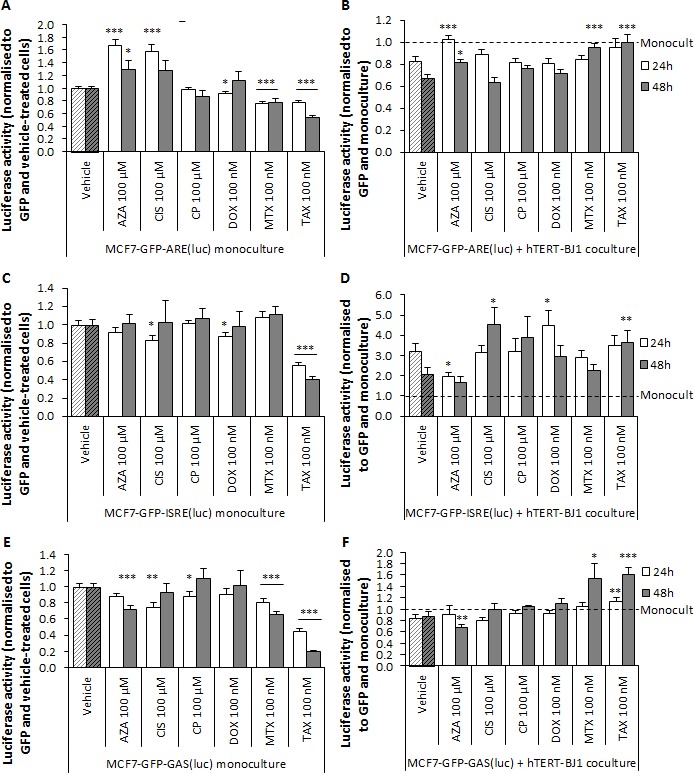
In response to chemotherapy, hTERT-BJ1 fibroblasts trigger antioxidant and immune response in MCF7-GFP cells Left panels. Chemotherapy effects on **A.** ARE, **C.** ISRE, and **E.** GAS signalling pathways in MCF7-GFP cells in monoculture after 24 and 48 h normalized to GFP fluorescence and vehicle-treated cells. Azathioprine and cisplatin showed increased antioxidant response in the monocultures, and doxorubicin, mitoxantrone and taxol, decreased. Most drugs inhibited interferon-mediated signalling in the MCF7-GFP monocultures. Mean ± SEM. Right panels. Chemotherapy effects on **B.** ARE, **D.** ISRE and **F.** GAS signalling pathways in MCF7-GFP cells in co-culture after 24 and 48 h normalised to GFP fluorescence and cells in monoculture. ARE signal was significantly activated in azathioprine, mitoxantrone and taxol co-cultures compared to vehicle-treated co-cultures. ISRE signalling was increased in cisplatin, doxorubicin and taxol co-cultures compared to vehicle-treated co-cultures. Finally, GAS signal was significantly activated in mitoxantrone and taxol co-cultures compared to vehicle-treated co-cultures. Mean ± SEM.

Using our co-culture system in a range of reporter cells, we pursued to identify the mechanism by which stromal cells promote chemoresistance, prompt metastasis or even *de novo* tumorigenesis. We hypothesised that therapy-induced stromal IL6 secretion would stimulate STAT3 in neighbouring cancer cells. Likewise, we assessed the effects of stromal cells on antioxidant (ARE), immune response (ISRE, GAS), or stem cell signalling (GLI, TCF/LEF, SMAD) in cancer cells when co-cultured with fibroblasts upon treatment. Therefore, several reporter MCF7-GFP cell lines were generated (Table 2), and cultured either as monolayers or co-cultured with fibroblasts, treated with chemotherapy, and assessed for luciferase activity. We first evaluated ARE-dependent signalling pathway. Azathioprine and cisplatin significantly increased luciferase signal in MCF7-GFP-ARE(luc) monocultures, whereas doxorubicin, mitoxantrone and taxol significantly decreased it (Figure 7A). ARE signalling was inhibited when fibroblasts were present in the culture. However, azathioprine, mitoxantrone and taxol co-culture treatments did not inhibit luciferase activity compared to vehicle, showing a reactivation of ARE signalling (Figure 7B).

Immune response is thought to be supressed in the body after anti-cancer treatment. Indeed, the drugs used in this study are also used as immunosuppressants. Therefore, we assessed interferon-mediated signalling, which was significantly inhibited by most drugs in MCF7 (Figure 7C, 7E) and hTERT-BJ1 monocultures (Figure S3B-S3C). Nevertheless, interaction with hTERT-BJ1 remarkably enhanced ISRE activation in MCF7 cells. Cisplatin, doxorubicin and taxol treatments further increased ISRE signalling in the co-cultures compared to vehicle, and azathioprine significantly decreased it (Figure 7D). The presence of hTERT-BJ1 in the culture reduced GAS signalling, yet carboplatin and doxorubicin did not inhibit GAS signalling and luciferase signal in mitoxantrone and taxol co-culture treatments was significantly higher than in the vehicle (Figure 7F). Thus, cancer cells in contact with stromal fibroblasts are able to elicit interferon-mediated signalling in response to chemotherapy.

**Figure 8:**
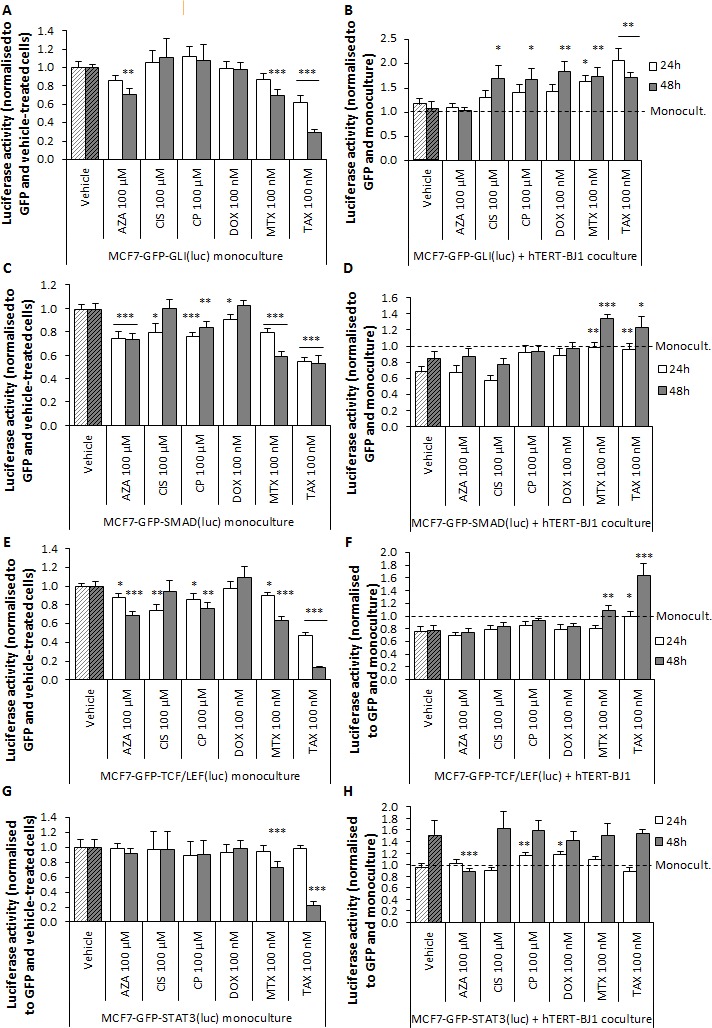
In response to chemotherapy, hTERT-BJ1 fibroblasts induce stemness-related pathways in MCF7-GFP cells Left panels Chemotherapy effects on **A.** GLI, **C.** SMAD, **E.** TCF/LEF and **G.** STAT3 signalling pathways in MCF7-GFP cells in monoculture after 24 and 48 h normalised to GFP fluorescence and vehicle-treated cells. Most drugs were able to inhibit GLI, SMAD and TCF/LEF signalling in the MCF7-GFP monocultures. Mitoxantrone and taxol also inhibited STAT3 in the monocultures. Mean ± SEM. Right panels Chemotherapy effects on **B.** GLI, **D.** SMAD, **F.**TCF/LEF and **H.** STAT3 signalling pathways in MCF7-GFP cells in co-culture after 24 and 48 h normalised to GFP fluorescence and cells in monoculture. All drugs except azathioprine activated GLI signalling pathway in the co-cultures compared to vehicle-treated co-cultures. SMAD and TCF/LEF signal was significantly activated also in mitoxantrone and taxol co-cultures compared to vehicle-treated co-cultures. Finally, carboplatin and doxorubicin were able to activate STAT3 signalling in the co-cultures. Mean ± SEM.

Finally, we determined the activation of stemness-related pathways in MCF7 cancer cells in synergy with stromal fibroblasts after chemotherapeutic exposure. Most drugs did not have an effect or inhibited all assessed pathways in the cancer cell monolayers (Figure 8A, 8C, 8E and 8G). In interaction with hTERT-BJ1 fibroblasts, Sonic hedgehog (Shh)/GLI signalling was not modified, TGFβ/SMAD and Wnt/TCF/LEF signalling pathways were decreased, and STAT3 signalling was activated at 48 h. However, in co-cultures, all drugs except azathioprine remarkably stimulated GLI signalling compared to vehicle, in MCF7 cancer cells (Figure 8B). Likewise, carboplatin and doxorubicin did not inhibit SMAD signalling as observed in the vehicle and luciferase activity of mitoxantrone and taxol co-culture treatments was significantly augmented. Similarly, carboplatin did not inhibit Wnt signalling, and mitoxantrone and taxol were able to stimulate TCF/LEF signalling compared to the vehicle (Figure 8D and 8F). Finally, carboplatin and doxorubicin reinforced STAT3 signalling after 24 h, whereas azathioprine inhibited it (Figure 8H).

In summary, chemotherapy inhibits most studied signalling pathways in MCF7 cancer cells. In contact with hTERT-BJ1 fibroblasts, though, MCF7 cancer cells establish a crosstalk with stromal cells as assessed by activation or inhibition of these pathways. That interaction, however, is altered upon treatment and stromal fibroblasts are able to stimulate antioxidant and immune response, and trigger various signalling pathways associated with tumour proliferation, survival and stemness in MCF7 cancer cells, providing new insights from which treatment failure and chemotherapy-induced tumorigenesis can be further investigated.

## DISCUSSION

### Chemotherapy promotes the catabolic tumour stroma phenotype

In this study we show for the first time the induction of a clear metabolic stress in stromal fibroblasts that favours glycolysis and lactate production to the detriment of mitochondrial metabolism in response to chemotherapy, phenotype previously defined as one of the hallmarks of the catabolic tumour stroma. Previous results in breast cancer imply that lactate secreted by stromal fibroblasts can be utilised as a mitochondrial fuel by the surrounding cancer cells [12, 23], and enables chemoresistance due to extracellular acidification [24]. Moreover, lactate administration increases the number of metastasis *in vivo* and stimulates migration [12] and expression of stemness-related genes in breast cancer cells *in vitro*. These gene signatures can in turn predict recurrence and metastasis in breast cancer [25].

Our results demonstrate that chemotherapy can stimulate HIF, NFkB, SMAD, STAT3 and JNK/AP1 stress-linked signalling pathways, and are consistent with previous studies showing activation of these signalling pathways due to oxidative stress by anticancer therapies also in healthy cells [6, 13, 32]. Oxidative stress in the stroma is generally caused by cancer cells at the onset of their metabolic synergy [6, 26] and promotes CAF transformation in the stromal compartment and invasiveness in breast cancer cells [15]. HIF1, NFkB, JNK, STAT3 and TGFβ activation in the stroma occurs with increased catabolism, which in turn can promote cancer proliferation [14, 16, 27-30] and can cause immunosuppression and angiogenesis, making cancer more invasive [31].

Exposure to chemotherapy also induced the transformation of stromal fibroblasts into myofibroblasts, another feature of the catabolic tumour stroma. It has been previously shown that the presence of fibrotic lesions actually enhances the risk of developing cancer [33], pointing at CAF transformation as a feature that can lead to *de novo* tumorigenesis.

In addition we detect increased autophagy, ketone production and senescence in chemotherapy-treated stromal fibroblasts. Autophagic hTERT-BJ1 fibroblasts, generated by over-expression of autophagy promoters, such as cathepsin B, and senescent hTERT-BJ1 fibroblasts, generated via over-expression of CDK inhibitors, such as p21, are able to enhance tumour growth and metastasis when co-injected with breast cancer cells *in vivo* [34, 35]. Moreover, ketogenic fibroblasts stimulate mitochondrial biogenesis in the neighbouring cancer cells [36], and up-regulation in ketogenic enzyme expression has recently been correlated with therapy failure [37]. Finally, systemic ketogenesis and high stromal senescence are commonly associated with tumorigenesis [38, 39]. Likewise a chronic inflammatory microenvironment also contributes to all tumorigenic stages such as the induction of neoplastic-related mutations, resistance to apoptosis or metastasis [40, 41]. The IL6/STAT3 pathway is particularly important in inflammation-related tumorigenesis [42]. Cancer cells induce NFkB signalling in tumour-associated macrophages (TAMs), triggering their IL6 secretion, which in turn stimulates the neoplastic proliferation via STAT3 activation [43]. Consistent with that, and with the fact that chemotherapy is associated with up-regulation of inflammation markers and stress response genes in healthy tissues [44, 45], we observed that chemotherapy-damaged fibroblasts trigger NFkB and other stress-induced pathways and promote the secretion of IL6, energy-rich metabolites and possibly other ligands, which have the potential to succour cancer cells in their battle for survival, in their purpose to metastasise, or even through which partially-transformed epithelial cells may be more susceptible to become fully malignant, giving rise to a new cancer.

### Stromal metabolic stress enhances stemness, antioxidant and immune response in cancer cells. Is the catabolic stroma able to persuade partially transformed cells into becoming fully malignant?

We finally sought to identify the mechanism by which stromal cells may promote chemoresistance, metastasis or *de novo* tumorigenesis using our co-culture system. Strikingly, Shh signalling was predominantly activated in breast cancer cells in contact with stromal cells in response to chemotherapy, as well as Wnt, STAT3 and TGFβ pathways, which are involved in progenitor and stem cell renewal, metabolism [46], metastasis and chemoresistance [47]. Abnormal activation of Shh, Wnt and TGFβ signalling is found in many types of cancer [48, 49]. In fact, *de novo* mutational activation of Shh pathway by itself can result in cancer [49], and it is also involved in the synergy of cancer and stromal cells, as stromal hh production stimulates neoplastic proliferation and metastasis [50], and tumour-secreted hh ligands in turn induce CAF transformation generating a tumour-promoting microenvironment [51]. Moreover, these pathways are involved in cellular metabolism. When STAT3 is found in the mitochondria enhances oxidative phosphorylation, encouraging the growth of cancer cells when over-expressed [52]. Wnt signalling also increments oxidative phosphorylation in breast cancer cells [53]. Interestingly, conditioned media from primary tumour fibroblasts or co-cultures with fibroblasts reduces chemotherapy efficacy due to improved mitochondrial function in the cancer cells [3], implying that upon therapy stromal fibroblasts may support cancer cells in their struggle for survival via stimulation of these stemness pathways.

In the present study, we also observe that in response to chemotherapy, Nrf1 and Nrf2-mediated signalling is reactivated in MCF7 cancer cells in contact with stromal fibroblasts. Several studies show constitutive up-regulation of Nrf2 in many cancers, which provides a growth advantage for cancer cells by protecting them from anticancer agents [54, 55].

Finally, in response to chemotherapy we detect that cancer cells in contact with stromal fibroblasts are able to elicit interferon-mediated signalling. In fact, exposure to chemotherapy *in vivo* induces recruitment of monocytic cells and macrophages to tumours, which is associated with cancer cell survival and poor therapy responsiveness [56, 57]. TAMs gather in the tumour microenvironment in response to cytokines such as Csf1, a STAT1 target gene. Indeed, *Stat1*-null animals show reduced macrophage infiltration [58], and in agreement with our findings, breast cancer microarray analyses indicate that high mRNA expression levels of *Stat1* and its target genes belonging to the interferon-mediated signalling correlate with poor drug response and infiltration of immune cells, in particular TAMs [59]. Hence, the response to a same stimulus is cell context-dependent and different microenvironment components participate in the development of malignancy and chemoresistance. Likewise, not all drugs induce an identical behavioural pattern. The diverse nature of the anti-tumour agents, and the doses and the length of the treatments selected for the study may account for these differences.

**Figure 9:**
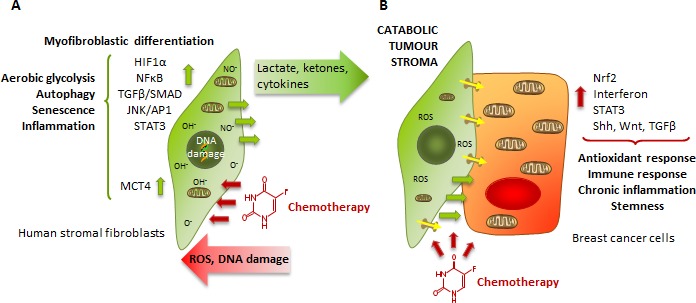
Chemotherapy induces the catabolic tumour stroma phenotype, which in turn activates antioxidant response, immune response and stemness in cancer cells **A.** Chemotherapy-induced DNA damage generate activation of HIF1, NFkB, TGFβ, STAT3 and JNK/AP1 signalling pathways in stromal cells, which stimulates their differentiation into CAFs, a switch to aerobic glycolysis and mitochondrial dysfunction, autophagy and senescence, release of inflammation cytokines and inhibition of interferon-mediated signalling. **B.** In contact with these catabolic fibroblasts, cancer cells react to chemotherapy by activating antioxidant and immune response signalling, and by activating stemness.

So far, very little is known about the role of healthy cells in the emergence of therapy-related malignancies. Initiation of tumorigenesis by accumulation of genetic changes is not sufficient for a tumour to develop, and requires the emergence of a reactive microenvironment, which via metabolic alterations and inflammation, contributes to the progression of the tumour and leads to treatment failure [1]. Therefore, cancer initiates in the tumour cell but gradually and systemically disseminates driving catabolism in healthy tissues. The mechanisms by which stromal metabolic stress controls the evolution of malignancy or even the susceptibility of a premalignant cell to become fully malignant are not understood. One piece of evidence shows that pre-treatment of tumour-free mice with a single dose of doxorubicin is sufficient to stimulate lung carcinoma cell engraftment and elevate the mitogenic activity of the serum of treated animals [9]. Here, we propose a new model by which upon treatment, stromal cells acquire a catabolic state that leads to an autophagic, nutrient-rich, senescent, pro-inflammatory microenvironment (Figure 9A), the ideal niche to encourage carcinogenesis by stimulating stemness in therapy-damaged pre-malignant epithelial cells and by increasing inflammation and immune cell infiltration, which in turn could further promote malignancy in these cells (Figure 9B). Nevertheless, given that successfully treating the primary cancer should always be a priority, targeting stromal cells in combination with conventional chemotherapy may help disrupting these ill-behaved microenvironments, improving treatment efficacy and avoiding future malignancies.

## MATERIALS AND METHODS

### Cell culture

Human foreskin fibroblasts immortalised with the human telomerase reverse transcriptase (hTERT-BJ1), and MCF7 breast cancer cells were purchased from ATCC and maintained in DMEM (D6546, Sigma) supplemented with 10% fetal bovine serum (F7524, Sigma), 100 units/ml of penicillin, 100 μg/ml, streptomycin (P0781, Sigma) and 1% Glutamax (#35050087, Life Technologies). For all experiments, cells were incubated in a 5% CO_2_ atmosphere at 37°C.

### Chemotherapeutical agents

Azathioprine, carboplatin, cisplatin, cyclophosphamide, doxorubicin, 5-fluorouracil, gemcitabine, methotrexate, 6-mercaptopurine, mitoxantrone, 6-thioguanine and taxol were used in this study. See Table 1 for details. Untreated and vehicle-treated cells were analysed in all experiments.

### Sulforhodamine b (SRB) assay

SRB (S9012, Sigma) measures total biomass by staining cellular proteins. After treatment, cells were fixed in 10% tricloroacetic acid (T9159, Sigma) for 1h at 4°C, stained with SRB (S9012, Sigma) for 15 minutes, and washed 3 times with 1% acetic acid (27225, Sigma). The incorporated die was solubilized with 10 mM Tris Base, pH 8.8 (T1503, Sigma). Absorbance was spectrophotometrically measured at 562 nm in a FluoStar Omega plate reader (BMG Labtech). Background measurements were subtracted from all values.

### L-lactate assay, β-hydroxybutyrate assay, glucose consumption

1.5 × 10^5^ hTERT-BJ1 cells per well were seeded in a 12 well plate. When cells were attached, drug treatments were added in triplicate for 48 or 72 h. Media was then collected. L-lactate was measured using the L-Lactate Assay Kit (735-10, Trinity Biotech) and β-hydroxybutyrate (β-HB) was determined with the β-HB Assay Kit (K632, Biovision), according to the manufacturer. L-lactate and β-HB production were calculated by subtracting the levels of L-lactate or β-HB in complete media from those in each sample. Glucose concentration was quantified using a FreeStyle Optium Glucose Meter (Abbott). Glucose consumption was calculated by subtracting the levels of glucose in each sample from those in complete media. For all assays, viable cells in each well for each time point were counted using Trypan Blue (T8154, Sigma) and used to normalize all results.

### ATP content

ATP content was measured using CellTiter-Glo (G7570, Promega) and ATP standards (A1852, Sigma). 2 × 10^4^ hTERT-BJ1 cells were seeded in black-walled 96 well plates. When cells were attached, drug treatments were added for 24, 48 or 72 h. Six replicates were used for each condition. Media was removed and CellTiter-Glo Assay was performed according to manufacturer's instructions. Light signal was acquired in the Xenogen VivoVision IVIS Lumina (Caliper Life Sciences). Results were normalised by SRB staining. ATP levels were calculated by extrapolating from the standard curve.

### Extracellular flux analysis

Extracellular acidification rate (ECAR) and oxygen consumption rate (OCR) were measured in a XF96 Extracellular Flux Analyzer (Seahorse Biosciences). 1.5 × 10^4^ hTERT-BJ1 cells per well were seeded in XF96 plates and incubated with complete medium. When cells were attached, drug treatments were added. Six replicates were run for each condition. After 72 h of drug treatments, un-buffered DMEM XF medium supplemented with 2 mM glutamine (pH 7.4) was added to the cells, and placed in a 37°C CO_2_-free incubator for 1h. 10 mM glucose, 1 μM oligomycin and 100 mM 2-deoxyglucose (2-DG) were injected into the media at different time points and ECAR was measured (Figure S2A). Likewise, ECAR and OCR were quantified using un-buffered DMEM XF medium supplemented with 2 mM glutamine, 2 mM sodium pyruvate and 10 mM glucose (Figure S2B). Cells were stained with SRB to normalise results. All parameters were calculated according to manufacturer.

### Levels of reactive oxygen species, autophagic vesicles and senescence

For all assays, 1.5 × 10^5^ hTERT-BJ1 cells per well were seeded in 12-well plates. When cells were attached, drug treatments were added for 24, 48 or 72 h in triplicate. Reactive oxygen species (ROS) production was measured using CM-H_2_DCFDA (C6827, Invitrogen). Cells were incubated for 20 min at 37°C with 1 μM CM-H_2_DCFDA diluted in PBS, and then placed in complete media for 20 min at 37°C in the dark, to render the die fluorescent, according to the manufacturer. Autophagy Detection Kit (ab139484, Abcam) was used to detect autophagic vesicle production. Cells were trypsinized and incubated for 30 min at RT in the dark with a solution containing 1:1000 of Green Detection Reagent and 1:1000 of Hoechst in phenol-red free DMEM (D5921, Sigma) supplemented with 5% FBS, according to the manufacturer. Senescent cells were detected using the fluorescent senescence-associated β-galactosidase (SA-β-gal) activity marker C_12_FDG (F1930, Molecular Probes). Cells were trypsinized and incubated with 2 mM of FDG substrate at 37°C for 1 minute. Staining was terminated by hypotonic shock by diluting the cells into ice-cold isotonic medium, according to the manufacturer.

ROS signal, autophagy vesicle signal, and SA-β-gal activity signal were quantified as mean fluorescent intensity of the viable cell population in a BD LSRII flow cytometer (BD Bioscience). Results were analyzed using FlowJo software.

### Western blotting

1 × 10^6^ hTERT-BJ1 fibroblasts were seeded in 10 cm dishes. When cells were attached, drug treatments were added for 48 h. Cells were lysed in RIPA lysis buffer (R0278, Sigma) containing proteinase inhibitors (05 892 970 001, Roche) and kept at 4°C for 20 minutes with rotation. Lysates were cleared by centrifugation for 10 minutes at 10,000 xg and supernatants were collected. Equal amounts of protein lysate, as determined by using the BCA protein assay kit (23225, Pierce) were diluted in SDS sample buffer and dry-boiled for 5 minutes before being separated by SDS-PAGE using 4-20% gels (456-1094, Biorad). Samples were then blotted onto nitrocellulose membranes (170-4159, Biorad), blocked in 5% milk in TBS-Tween 20 (P9416, Sigma) for 1h and probed with antibodies against α-tubulin (ab4074, Abcam), p-SAPK/JNK (T183-Y185) (4668S, Cell Signalling), P-c-Jun (S63) (926L1, Cell Signalling), total JNK (9252S, Cell Signalling), total c-Jun (2315S, Cell Signalling), p53 (OP43, Calbiochem), p21 (sc-756, Santa Cruz), and α-SMA (sc-53142, Santa Cruz). Bound antibodies were detected using a horseradish peroxidase-conjugated secondary antibody (ab6789 and ab6721, Abcam) and signal was obtained using Supersignal West Pico chemiluminiscent substrate (34087, ThermoScientific). Pictures were taken in a ChemiDoc XRS with Image Lab Software (BioRad).

### Immunofluorescence

1.5 × 10^5^ hTERT-BJ1 fibroblasts per well were seeded in coverslips in 12 well plates. When cells were attached, drug treatments were added for 72 h in triplicate. Cells were fixed with 2% paraformaldehyde (28908, Thermo Scientific) in PBS for 20 minutes at RT, permeabilised in −20°C-cold methanol for 5 minutes at 4°C, quenched with 50 mM NH4Cl in PBS for 10 minutes, rinsed and blocked with IF buffer consisting of 1% BSA (A3608, Sigma) plus 0.1% Tween 20 (P9416, Sigma) in PBS for 1h. Cells were then incubated for 1h with the MCT4 primary antibody (sc-50329, Santa Cruz). The fluorescent secondary antibody was added for 30 minutes. Cells were counterstained with DAPI and samples were mounted using Prolong Gold anti-fade reagent (P36934, Invitrogen). Immunofluorescence pictures were taken in a Leica gated Stimulated Emission Depletion Microscopy (gSTED) with additional confocal and multi-photon illumination (room rg106).

### Cytokine antibody array

Cytokine human membrane antibody array (Abcam, ab133998) was used as to screen cytokine secretion (N=1). 1 × 10^6^ hTERT-BJ1 fibroblasts were seeded in 10 cm plates until cells were attached and doxorubicin or vehicle were added to the cells for 72 h. Culture media was then placed on the membrane. The dot blot array was developed using Supersignal West Pico chemiluminiscent substrate. Pictures were taken in the ChemiDoc XRS with Image Lab Software and ImageJ software was used to quantify the intensity of the dots. Internal membrane loading controls were used to normalise results.

### Interleukin 6 ELISA

Interleukin 6 (IL6) concentration was determined using IL6 Human ELISA Kit (ab100572, Abcam). 3 × 10^5^ hTERT-BJ1 fibroblasts were seeded in 6 well plates. When cells were attached, drug treatments were added for 72 h in duplicate. Equal amounts of culture media, as determined by using the BCA protein assay kit, were loaded into the ELISA plate and incubated with the antibody overnight at 4°C with gentle shaking. Standards and media samples were run in parallel. Absorbance was spectrophotometrically measured at 450 nm in a FluoStar Omega plate reader (BMG Labtech). Background measurements were subtracted from all values. Finally, IL6 levels were calculated by extrapolating from the standard curve.

### Lentivirus infection

The Cignal Lenti reporter assay (luc) was used to monitor the activity of several signalling pathways in either hTERT-BJ1 fibroblasts or MCF7-GFP cells (see Table 2 for details). Viral particles diluted 1:10 in complete media containing polybrene (sc-134220, Santa Cruz) were added to the cells. Puromycin treatment (P9620, Sigma) was added 48 h later in order to stably select infected cells.

### Luciferase assay

Luciferase Assay System (E1501, Promega) was performed according to manufacturer's instructions. 2 × 10^4^ hTERT-BJ1 fibroblasts were seeded in black-walled 96 well plates. When cells were attached, drug treatments were added for 24, 48 and 72 h. Six replicates were used for each condition. After treatment, Luciferase Assay was performed according to manufacturer's instructions and light signal was acquired in the Xenogen VivoVision IVIS Lumina. Results were normalised by SRB staining. Likewise, 6 × 10^3^ MCF7-GFP cells were seeded in black-walled 96-well plates as single cultures or in combination with 6 × 10^3^ hTERT-BJ1 fibroblasts. When cells were attached, drug treatments were added for 24 and 48 h in quadruplicate. After treatment, GFP fluorescence was measured in the plates and Luciferase Assay was performed as before. Results were normalised by GFP fluorescence.

### Statistical analyses

Student's t test was used for statistical comparison of two groups. All data are reported as mean ± standard deviation of the mean (SEM). All experiments were repeated at least two times with reproducible results. *P* values lower than 0.05 were considered significant (*P < 0.05, **P < 0.01, ***P < 0.001). Microsoft Excel was used to produce all graphs.

## SUPPLEMENTARY MATERIAL, FIGURES AND TABLES



## Abbreviations

AZA: azathioprine
CIS: cisplatin
CP: carboplatin
DOX: doxorubicin
MTX: mitoxantrone
TAX: taxol
CAF: cancer-associated fibroblast
TAM: tumour-associated macrophage
β-HB: β-hydroxybutyrate
ECAR: extracellular acidification rate
OCR: oxygen consumption rate
2-DG: 2-deoxyglucose
MCT4: monocarboxylate transporter 4
SASP: senescence-associated secretory phenotype
IL6: interleukin 6
ROS: reactive oxygen species
SA-β-gal: senescence-associated-β-galactosidase
αSMA: α-smooth muscle actin
