# Supplementary material for: Chemotherapy induces the cancer-associated fibroblast phenotype, activating paracrine Hedgehog-GLI signalling in breast cancer cells
Source: Oncotarget. 2015 Apr 14;6(13):10728–45. doi: 10.18632/oncotarget.3828 (PMC4484415; doi:10.18632/oncotarget.3828)
Supplement: Supplementary file 1 [file oncotarget-06-10728-s001.pdf]

Chemotherapy induces the cancer-associated fibroblast phenotype, activating paracrine Hedgehog-GLI signalling in breast cancer cells

Supplementary Material

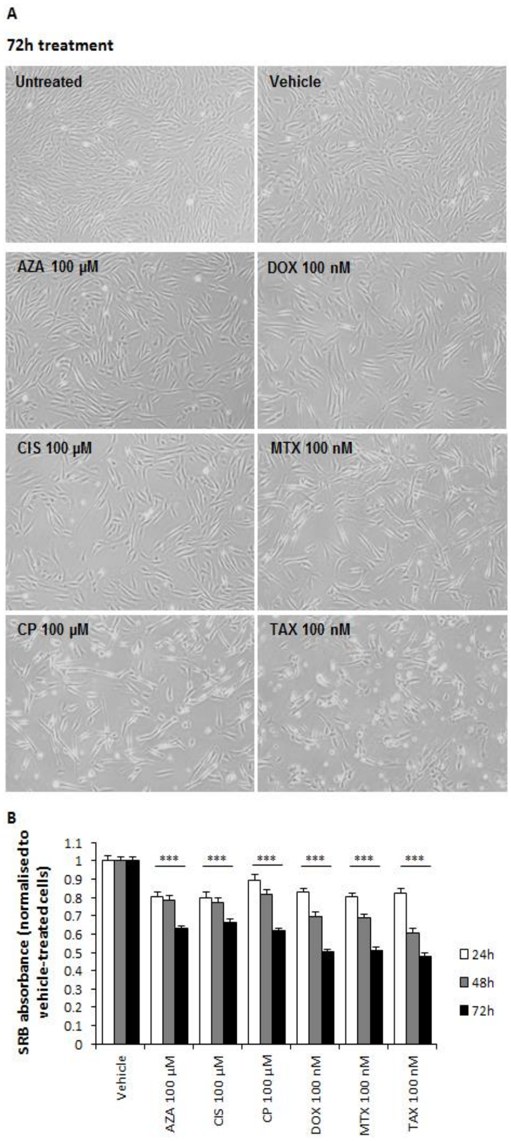

Figure S1: Azathioprine, cisplatin, carboplatin, doxorubicin, mitoxantrone and taxol were the drugs used in this study

A) Representative pictures of hTERT-BJ1 fibroblasts treated for 72 h with the different drugs. Vehicle-treated and untreated cells are also shown

B) SRB staining of hTERT-BJ1 fibroblasts treated with chemotherapy for 24, 48 and 72 h. All selected concentrations were sub-lethal and induced a similar loss in cell viability. Mean ± SEM

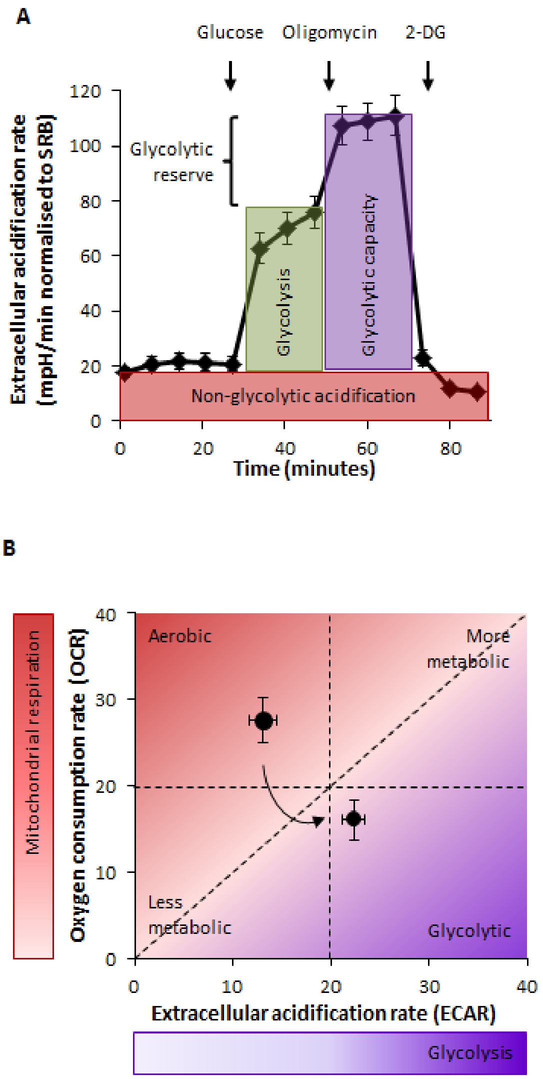

Figure S2: Extracellular flux analysis

A) The pH of the extracellular media is monitored throughout the experiment. Glucose addition produces a rapid rise in ECAR, which is attributable to glycolysis. Addition of oligomycin blocks mitochondrial ATP synthesis, further increasing the glycolytic rate of proliferative cells to fulfil their metabolic demands. Finally, addition of 2-DG completely blocks glycolysis

B) OCR versus ECAR in the presence of glucose in the media indicates the metabolic state of cells in culture

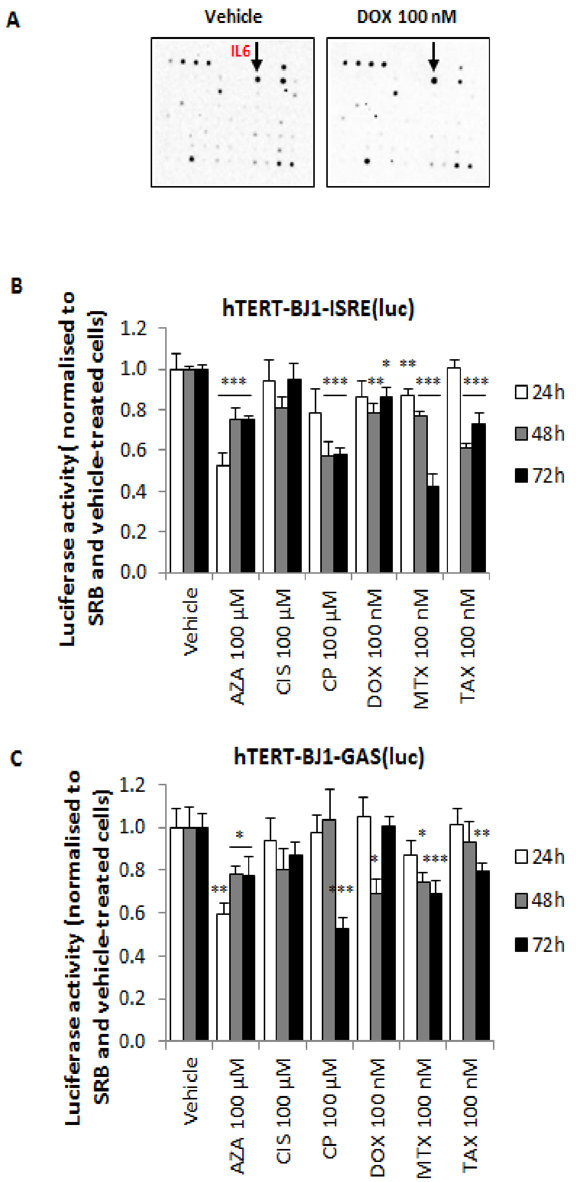

Figure S3: Chemotherapy induces IL6 secretion and inhibits interferon-mediated signalling in hTERT-BJ1 fibroblasts

A) Cytokine membrane antibody array indicated an up-regulation of the amounts of the IL6 cytokine in media of hTERT-BJ1 fibroblasts treated for 72 h with doxorubicin compared to vehicle-treated cells

B) Significant inhibition of ISRE and **C)** GAS signalling by chemotherapy after 24, 48 and 72 h normalised to SRB and vehicle. All drugs except cisplatin decreased ISRE and GAS signalling. Mean ± SEM

Table S1: L-Lactate secretion by hTERT-BJ1 cells treated with chemotherapy for 48 and 72 h normalised to vehicle-treated cells. Fold increases higher than 1.5 are indicated with +, higher than 2, with ++, and higher than 5 with +++.

Fold increase in L-Lactate production		48 h	72 h
Vehicle		1.00	1.00
Azathioprine (AZA)	10 μM	-	-
	100 μM	-	-
	500 μM	++	++
Carboplatin (CP)	10 μM	+	-
	100 μM	++	++
	1000 μM	++	+++
Cisplatin (CP)	1 μM	-	-
	10 μM	+	-
	200 μM	+	++
Cyclophosphamide (CPA)	10 μM	-	-
	100 μM	-	+
	1000 μM	-	-
Doxorubicin (DOX)	0.001 μM	-	-
	0.01 μM	+	-
	0.1 μM	++	+++
5-Fluorouracil (5-FU)	1 μM	-	-
	10 μM	-	-
	100 μM	-	-
Gemcitabine (GC)	0.01 μM	-	-
	0.1 μM	-	-
	1 μM	+	-
Methotrexate (MET)	10 μM	-	-
	100 μM	-	+
	1000 μM	++	+
6-Mercaptopurine (6-MP)	10 μM	+	-
	100 μM	+	+
	1000 μM	++	++
Mitoxantrone (MTX)	0.001 μM	+	-
	0.01 μM	++	+
	0.1 μM	+	+
Taxol (TAX)	0.001 μM	-	-
	0.01 μM	+	+
	0.1 μM	++	++
6-Thioguanine (6-TG)	10 μM	-	+
	100 μM	+	-
	1000 μM	+	-

Table S2. β-HB secretion by hTERT-BJ1 cells chemotherapy-treated for 72 h normalised to vehicle-treated cells. Fold increases higher than 1.5 are indicated with +, higher than 2, with ++, and higher than 5 with +++.

Fold increase in β-HB production		72 h
Vehicle		1.00
AZA	10 µM	+
	100 µM	++
	1000 µM	+++
CP	10 µM	-
	100 µM	++
	1000 µM	++
CIS	1 µM	-
	10 µM	+
	200 µM	+++
CPA	10 µM	-
DOX	0.001 µM	++
	0.01 µM	++
	0.1 µM	+++
5-FU	1 µM	-
	10 µM	-
	100 µM	-
GC	0.01 µM	++
	0.1 µM	++
	1 µM	++
MET	10 µM	+
	100 µM	+
	1000 µM	++
6-MP	10 µM	-
	100 µM	+
	1000 µM	++
MTX	0.001 µM	+
	0.01 µM	+
	0.1 µM	++
TAX	0.001 µM	+
	0.01 µM	++
	0.1 µM	+++
6-TG	10 µM	-
	100 µM	-
	1000 µM	-
